# Transforming hypoglycemia prediction in adult type 1 diabetes: a systematic review and meta-analysis for precision care

**DOI:** 10.1515/biol-2025-1325

**Published:** 2026-05-11

**Authors:** Qiang Zhang, Haojie Zhou, Xiaoli Zhu, Ruonan Lin, Liping Hu, Guniqing Zhu

**Affiliations:** School of Nursing, Dali University, Yunnan, China, 671000; Department of Cardiovascular Medicine, The Second Affiliated Hospital of Chongqing Medical University, Chongqing, China, 400010; Department of Endocrinology, The First Affiliated Hospital of Dali University, Yunnan, China, 671000; First Clinical Medical College, Southern Medical University, Guangdong, China, 510515

**Keywords:** type 1 diabetes, systematic review, meta-analysis, hypoglycemia, machine learning algorithms, predictive models

## Abstract

Type 1 diabetes mellitus (T1DM) patients require lifelong insulin therapy; however, iatrogenic hypoglycemia remains a major clinical challenge, with high incidence in adults. This study evaluated the performance, methodological rigor, and clinical utility of hypoglycemia risk prediction models for adult T1DM patients to inform evidence-based risk management strategies. Following Cochrane framework and PRISMA guidelines, 18 studies were identified. Data extraction and bias assessment were conducted using the PROBAST tool. The mean area under the curve (AUC) across individual models was 0.85. Meta-analysis of AUC values revealed a pooled AUC of 0.88 (95 % CI: 0.88–0.89), indicating moderate-to-good predictive accuracy. Substantial heterogeneity was observed (*I* ^2^ = 99.82 %, *P* < 0.001), mainly due to differences in prediction time windows, data sources, and validation strategies. Most studies (88.9 %) showed high or unclear risk of bias, and clinical applicability was limited, with only one study meeting criteria for low bias and high applicability. While existing models show moderate predictive performance, significant methodological limitations exist. Future research should focus on optimizing study design, conducting multi-center investigations, developing interpretable AI, standardizing validation protocols, and integrating these models into clinical practice to improve hypoglycemia management.

## 1 Introduction

Patients with type 1 diabetes mellitus (T1DM) rely on continuous exogenous insulin replacement therapy to maintain metabolic homeostasis [1]. However, iatrogenic hypoglycemia remains a major clinical challenge that compromises disease management and patient prognosis [2]. Epidemiological evidence indicates that compared with pediatric and adolescent populations, adult T1DM patients face more intricate lifestyle management dilemmas, a higher prevalence of multi-system comorbidities, and altered insulin sensitivity. These factors significantly increase the risk of hypoglycemia in this population. Among adults with T1DM, the incidence of hypoglycemia is as high as 73.3 %, with severe hypoglycemic events occurring at an approximate rate of 4.9 %. Such adverse events not only induce cognitive dysfunction and acute cardiovascular events but also pose a potential risk of mortality, underscoring the urgency of optimizing hypoglycemia risk management in adult T1DM patients [3].

Although conventional management strategies, such as continuous glucose monitoring (CGM), individualized dietary counseling, and insulin dose adjustments, can help mitigate the risk of hypoglycemia, some patients still experience asymptomatic hypoglycemia. Asymptomatic hypoglycemia is defined as a blood glucose level ≤3.9 mmol/L without typical sympathetic nervous system activation symptoms [4]. Recurrent hypoglycemic episodes diminish the body’s ability to sense low glucose levels, thereby elevating the risk of severe adverse outcomes [5]. Notably, asymptomatic hypoglycemia is clinically significant due to its insidious nature. It silently induces cognitive impairment by damaging glucose-dependent central nervous system cells, triggers silent cardiovascular risks including myocardial ischemia, and raises severe hypoglycemia and fall risks especially in elderly adult T1DM patients with comorbidities to worsen prognosis [6], 7]. However, these strategies fail to address the core pathological mechanisms underlying T1DM progression, namely inflammation and oxidative stress, which accumulating studies have identified as closely intertwined with hypoglycemia in T1DM. Beyond iatrogenic factors such as inappropriate insulin administration, dysregulated inflammatory responses and excessive oxidative stress further contribute to the occurrence and recurrence of hypoglycemia.

T1DM is characterized by chronic inflammation and excessive production of reactive oxygen species (ROS), which not only mediate the destruction of pancreatic β-cells but also impair insulin sensitivity and metabolic regulation in target tissues such as skeletal muscle, liver, and adipose tissue. Streptozotocin-induced diabetic models have confirmed that elevated ROS levels in pancreatic islets are associated with impaired insulin secretion, which has highlighted oxidative stress as a key disruptor of glucose homeostasis. Hydrogen sulfide (H_2_S), an endogenous gasotransmitter with inherent antioxidant and anti-inflammatory properties, has been shown to protect against hepatotoxicity and pancreatic damage by inhibiting the TLRs-JNK/NF-κB inflammatory pathway, underscoring the critical role of dysregulated inflammatory responses in metabolic instability [8]. Long non-coding RNA H19, whose expression is downregulated in the skeletal muscle and liver tissues of diabetic individuals, regulates insulin sensitivity by interacting with heterogeneous nuclear ribonucleoprotein A1 to modulate the expression of fatty acid oxidation-related genes [9]. Its dysregulation disrupts the balance of glucose-lipid metabolism, further exacerbating metabolic instability related to hypoglycemia [10].

Complementary interventions targeting these pathological pathways have yielded promising preclinical results, with key strategies directly targeting the core defects of T1DM-related hypoglycemia. Chlorella supplementation alleviates oxidative stress and promotes pancreatic β-cell regeneration [11]. The combined application of bone marrow mesenchymal stem cells and pioglitazone, a peroxisome proliferator-activated receptor γ agonist, improves insulin resistance and upregulates the key metabolic mediators PGC-1α and GLUT4 [12]. Vitamin E, a lipid-soluble chain-breaking antioxidant, reduces blood glucose levels and enhances endogenous antioxidant enzyme activities [13], 14]. The results confirm the intricate interplay between oxidative stress, organ dysfunction, and metabolic dysregulation in predisposing to hypoglycemia. Relevant translational patents focusing on targeted antioxidant agents, anti-inflammatory agents, and β-cell protective agents have laid the foundation for clinical transformation of these strategies.

Previous studies have identified factors such as long diabetes duration and elevated glycosylated hemoglobin levels as closely associated with the risk of hypoglycemia in adult T1DM patients [15]. However, traditional risk assessment approaches based on clinical experience are plagued by high subjectivity and limited predictive accuracy [16]. In recent years, advances in artificial intelligence (AI) have facilitated the development of machine learning (ML)-based prediction models, providing new avenues for precise the risk of hypoglycemia stratification and management [17]. Notably, specific ML/AI techniques have driven methodological innovation in this field. For example, time-series analysis of CGM data enables accurate capture of dynamic glucose fluctuations and noise reduction [18]. Deep learning techniques extract multi-dimensional features from glucose and insulin sequences to improve prediction accuracy [19]. Ensemble methods such as XGBoost integrate hundreds of predictive factors to enhance the robustness of severe hypoglycemia prediction [20]. International studies have demonstrated that these models can significantly improve the accuracy and timeliness of hypoglycemia prediction by integrating multi-dimensional data, including dynamic CGM features, insulin infusion patterns, and lifestyle-related metrics [21]. Importantly, the integration of ML-based hypoglycemia prediction models with translational patents has further strengthened the translational relevance of this field, bridging the gap between preclinical research, clinical practice, and industrial application.

Notably, the pathological mechanisms including inflammation, oxidative stress, and metabolic dysregulation elucidated, provide a critical theoretical basis for optimizing these ML-based prediction models. H19 dysregulation, oxidative stress markers such as reduced glutathione and thiobarbituric acid reactive substances, inflammatory pathways molecules (TLR2/4, NF-κB), and the expression of key metabolic mediators (PGC-1α, GLUT4) are closely correlated with metabolic instability [8], [9], [10, 12]. They offer potential biomarkers for refined the risk of hypoglycemia stratification. Furthermore, the hepatoprotective effect of H_2_S donors via TLRs-JNK/NF-κB inhibition, the β-cell protective role of chlorella, the metabolic regulatory effects of MSCs combined with pioglitazone, and the antioxidant capacity of vitamin E further support the integration of pathway-specific indicators into prediction frameworks [8], [12], [13], [14]. These indicators include serum vitamin E levels, glutathione S-transferase activity and GLUT4 expression. The integration of these indicators can enhance the performance of prediction frameworks. These pathway-specific indicators are closely linked to the targets of existing translational patents, also providing potential biomarkers for patent-protected intervention strategies and further highlighting the translational value of integrating preclinical mechanisms, prediction models, and patent technologies in hypoglycemia management. However, the integration of these pathway-specific indicators into existing prediction models remains understudied, further supporting the need for a systematic review and meta-analysis of current models.

Despite these advances, current hypoglycemia prediction models for adult T1DM patients exhibit significant heterogeneity in modeling approaches, validation cohorts, and clinical applicability. Many studies are limited by single-center data, which restricts the generalizability of the models. Furthermore, the lack of CGM data in most studies compromises their predictive ability for asymptomatic hypoglycemia. Additionally, the adaptability of these models to complex clinical scenarios remains unclear. More importantly, existing models often fail in clinical practice due to several critical limitations. Insufficient external validation in diverse populations leads to poor generalizability, overfitting to training data reduces the real-world performance, limited interpretability hinders clinicians’ trust and adoption, and poor integration into existing electronic health record (EHR) systems and clinical workflows hinders the translation of model predictions into timely clinical interventions. Therefore, this study aims to conduct a comprehensive systematic review and meta-analysis to evaluate the performance characteristics, methodological quality, and clinical utility of existing prediction models. The findings of this study are expected to provide an evidence-based foundation for optimizing the risk of hypoglycemia management strategies in adult patients with T1DM; crucially, improved prediction models can enhance patient quality of life by reducing fear of hypoglycemia and enabling independent daily activities, decrease hospitalization rates related to severe hypoglycemic events, and lower healthcare costs associated with emergency treatments and long-term complication management. Ultimately, this will improve their disease prognosis and quality of life.

## 2 Materials and methods

### 2.1 Problem formulation

This research rigorously adheres to the methodological framework established by the Cochrane systematic reviews [22]. The PICOTS model was employed to formulate clear, evidence-based research questions:

– **Population (P):** Adult patients (≥18 years) diagnosed with T1DM.
– **Index model (I):** Hypoglycemia risk prediction models specifically designed for adult T1DM patients. These models must include at least two predictors and have undergone statistical validation.
– **Comparator model (C):** Not applicable.
– **Outcome measure (O):** Incidence of hypoglycemic events (dichotomous outcome).
– **Timing (T):** Covers the entire period from model development to validation.
– **Setting (S):** Both clinical and community-based settings were included.

This study was conducted in strict accordance with the PRISMA statement and was prospectively registered with PROSPERO (registration number: CRD420251012275) to ensure transparency and reproducibility [23].

### 2.2 Search strategy

A comprehensive search was performed across eight Chinese and English databases. These databases included PubMed, Web of Science, Cochrane Library, Embase, CNKI, Wanfang Data, VIP Database, and the China Biomedical Literature Database (CBM). The search spanned from the inception of each database up to March 16, 2025, ensuring temporal comprehensiveness. The search query combined subject headings and free-text terms. For instance, in PubMed, the following search strategy was applied:

((Diabetes Mellitus, Type 1 [MeSH] OR Type 1 Diabetes [Title/Abstract]) AND (Hypoglycemia [MeSH] OR Hypoglycemia [Title/Abstract])) AND (predict [Title/Abstract] OR model [Title/Abstract] OR riskscore [Title/Abstract] OR AUC [Title/Abstract] OR nomogram [Title/Abstract]))*.

Additionally, a supplementary search was conducted by manually screening the references of the included studies using the literature traceability method. Grey literature sources, such as Baidu Scholar and Google Scholar, were also searched to ensure a comprehensive review. The search strategy was collaboratively developed by three investigators and implemented after pre-search testing to maintain an optimal balance between recall and precision.

### 2.3 Literature inclusion and exclusion criteria

#### 2.3.1 Inclusion criteria

– The study population consisted of adult patients (aged 18 years or older) diagnosed with T1DM.
– The study focused on developing or validating a hypoglycemia risk prediction model that incorporated at least two predictors.
– The primary outcome measured was the occurrence of hypoglycemic events (dichotomous outcome).
– The study design was a cohort study, case-control study, or randomized controlled trial.
– The publication language was Chinese or English.

#### 2.3.2 Exclusion criteria

– Studies involving non-adult T1DM populations.
– Studies lacking clear reporting on the development or validation of the predictive model.
– Studies with significant methodological flaws.
– Non-original research articles, including reviews, commentaries, conference abstracts, and case reports.
– Studies based on simulated data.
– Studies for which full-text articles were unavailable or that were duplicate publications.

### 2.4 Literature screening and data extraction

The literature selection and data extraction processes strictly followed the methodological standards of the Cochrane systematic review [22]. The detailed procedure was as follows: After deduplication using Zotero 7.0, two researchers trained in evidence-based medicine independently conducted an initial screening of titles and abstracts, excluding clearly irrelevant studies such as those not involving predictive models or not related to the T1DM population. The remaining articles underwent a second round of screening, and final inclusion was determined based on the predefined inclusion and exclusion criteria. Any discrepancies were resolved through arbitration by a third researcher with senior professional expertise.

Data extraction was performed using a structured table developed in accordance with the Cochrane Guidelines for Systematic Reviews of Prediction Models [22]. The table captured key elements including study characteristics (e.g., authors, publication year, study region), methodological details (e.g., study design, sample size, data sources), model features (e.g., predictors included, modeling algorithms, validation strategies), and performance metrics (e.g., AUC, sensitivity, specificity, calibration). To ensure accuracy, two researchers independently extracted the data, with subsequent data verification completed using the automatic verification function in Microsoft Excel 2023.

### 2.5 Risk of bias and applicability assessment of the included literature

In this study, we employed the internationally recognized Prediction Model Risk of Bias Assessment Tool (PROBAST) [24]. Two investigators trained in the PROBAST system independently conducted risk of bias (ROB) and applicability assessments. As the first dedicated assessment tool for predictive models endorsed by the Cochrane Collaboration, PROBAST has been validated across multiple fields in both diagnostic and prognostic prediction research [25]. The evaluation process rigorously adhered to the four core domains of PROBAST including participants, predictors, outcomes, and analysis along with its 20 signal questions. A three-tiered scoring system including “low risk”, “high risk”, or “unclear” was applied to assess the risk of bias, and a three-dimensional clinical relevance analysis encompassing participant, predictor, and predicted outcome was used to evaluate model applicability [26]. Discrepancies were resolved through discussion and when needed, adjudicated by a third researcher with senior professional expertise.

### 2.6 Statistical methods

#### 2.6.1 Data extraction and handling of missing data

A standardized data extraction table was utilized to organize the baseline characteristics, modeling methods, and performance indicators of the included studies. Studies were included only if they reported sufficient data to calculate effect sizes including AUC and 95 % confidence intervals (CIs). Studies with missing AUC values or incomplete performance metrics were excluded from quantitative synthesis. No imputation methods were used for missing outcome data in the primary studies. A dynamically updated database of study features was subsequently established.

#### 2.6.2 Classification of prediction models

Prediction models included in this review were classified according to modeling algorithms. The classification was applied in all subsequent quantitative and qualitative analyses. The AUC value served as the primary effect size, and both the AUC and its 95 % confidence interval (CI) were extracted from each study. Heterogeneity between studies was assessed using the *I* ^2^ statistic where an *I* ^2^ value > 50 % indicated significant heterogeneity and Cochran’s *Q* test with *P* < 0.10 considered indicative of heterogeneity. When *I* ^2^ was ≤50 % and the *Q*-test *p*-value was ≥0.10, effect sizes were pooled using a fixed-effect model. In cases of significant heterogeneity, a random-effects model was applied.

Meta-analysis of the AUC values was conducted using R software version 4.2.1 with the meta package. Forest plots were generated and pooled effect sizes were calculated. All statistical code and analysis scripts are available upon request to ensure full reproducibility in accordance with open science principles.

#### 2.6.3 Subgroup and sensitivity analyses

Planned subgroup analyses were performed according to model type, data source such as CGM versus non-CGM, and study geographic region. Sensitivity analyses were conducted to compare pooled results between studies with low risk of bias and those with high or unclear risk of bias.

#### 2.6.4 Publication bias

Publication bias was assessed with Egger’s test. Funnel plots were also constructed and visually inspected to supplement statistical testing for asymmetry.

## 3 Results

### 3.1 Literature screening process

This study meticulously developed a literature screening strategy in strict adherence to the PRISMA statement [23]. The process involved several steps: an extensive search across eight major Chinese and English databases, complemented by a search for grey literature. This yielded a total of 10,478 initial articles. Deduplication was performed using Zotero 7.0, resulting in 8,678 unique records for preliminary screening.

**Figure 1:**
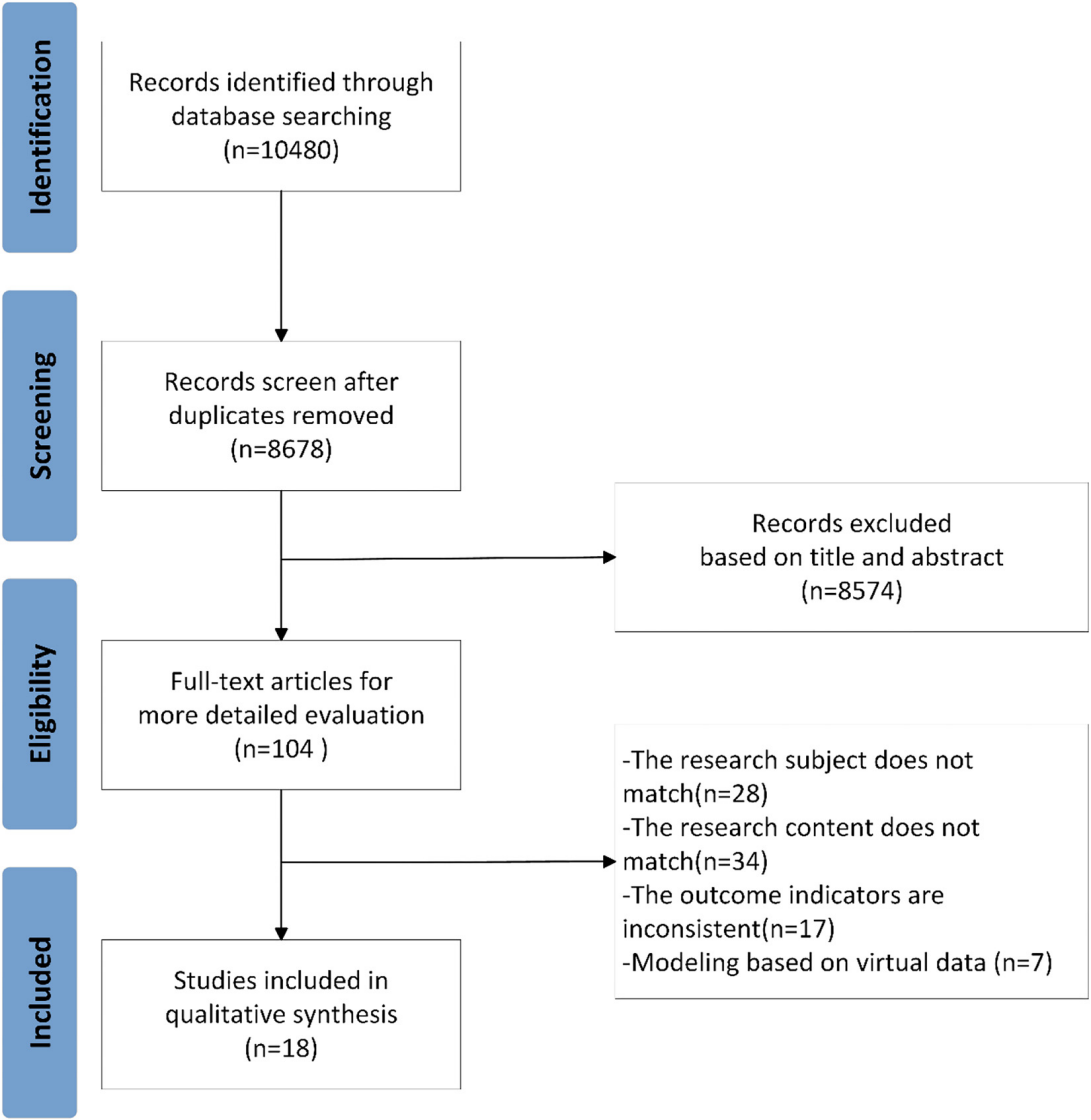
PRISMA flow diagram of literature screening.

Two investigators trained in evidence-based medicine independently conducted the initial screening by reviewing the titles and abstracts. Through this process, 8,574 studies that were clearly irrelevant were excluded. The remaining 104 full-text articles underwent a thorough re-screening based on pre-defined inclusion and exclusion criteria. Ultimately, 18 studies met the eligibility requirements and were included in the final analysis (Figure 1).

### 3.2 Basic characteristics of included studies

In this study, a total of 18 articles published between 2007 and 2025 were included [19], [27], [28], [29], [30], [31], [32], [33], [34], [35], [36], [37], [38], [39], [40], [41], [42], [43]. These studies originated from eight countries, including the United Kingdom, Spain, Denmark, and China. Retrospective studies comprised the majority (*n* = 11; 61.1 %) [19], 29], [34], [35], [36], [37], [38], [39], [40], [41, 43], followed by prospective studies (*n* = 6) [28], [30], [31], [32], [33, 42] and a single randomized controlled trial (*n* = 1) [27]. Sample sizes varied widely, ranging from 10 to 9,800 participants, with a median of 45.

Data sources were primarily centered on CGM (*n* = 12) [19], [27], [28], [29], [30], [31, 33], 35], [37], [38], [39, 43]. Among these, four studies also incorporated activity monitors [27], 30], 33], 39], while two studies integrated clinical records [31], 40]. There was notable heterogeneity in the prediction time windows: nighttime prediction accounted for the largest proportion (*n* = 7) [27], 29], 30], 34], 39], 41], 43], followed by exercise-specific prediction (*n* = 3) [28], 40], 42] and postprandial-specific prediction (*n* = 2) [37], 38].

Regarding study endpoint definitions, 15 studies (83.3 %) [19], [27], [28], [29], [30], [31, 33], [35], [36], [37], [38], [39], [40, 42], 43] defined hypoglycemia as a blood glucose level below 70 mg/dL (or 3.9 mmol/L). Two studies (11.1 %) [32], 34] used severe hypoglycemic events (requiring external intervention) as the endpoint, and one study did not clearly report a specific threshold [41]. All studies were conducted exclusively in adult T1DM populations (aged ≥18 years). Baseline characteristics are detailed in Table 1.

**Table 1:** Demographic and methodological characteristics of included studies (*n* = 18).

Author(s) (Year)	Region	Population	Age $\left(\mu \pm \overset{®}{x}\right)$	Sample size	Data source	Research type	Endpoint event(s)	Prediction time
Shao et al. (2024) [19]	China	T1DM	40.5 ± 13.02	17	CGM	Retrospective study	Hypoglycemia (blood glucose = 3.0–3.9 mmol/L or < 3.0 mmol/L)	30-min Prediction Window
Afentakis et al. (2025) [27]	The United Kingdom	T1DM	36.0 ± / 49.0 ± /	37	CGM, Activity Tracker, Smartphone Application	RCT	Hypoglycemia (blood glucose < 3.9 mmol/L for at least 20 consecutive minutes)	Nighttime
Bergford et al. (2023) [28]	Multicenter	T1DM	37.0 ± 14.00	459	CGM	Prospective study	Hypoglycemia (at least one CGM value < 3.9 mmol/L during exercise)	Exercise
Berikov et al. (2022) [29]	Russia	T1DM	36.0 ± /	406	CGM	Retrospective study	Hypoglycemia (interstitial glucose level remains below 3.9 mmol/L for at least 15 min)	Nighttime
Bertachi et al. (2020) [30]	Spain	T1DM	31.8 ± 16.80	10	CGM, Fitness Bracelet	Prospective study	Hypoglycemia (blood glucose < 3.9 mmol/L during sleep)	Nighttime
Cichosz et al. (2014) [31]	Denmark	T1DM	44.0 ± 15.00	10	CGM, Electrocardiogram (Lead II)	Prospective study	Hypoglycemia (blood glucose ≤ 3.9 mmol/L)	22-min Prediction Window
Cox et al. (2007) [32]	The United States	T1DM	40.7 ± 11.20	90	Patients’ Self-Monitored Blood Glucose Records	Prospective study	Severe hypoglycemia (impaired consciousness or inability to handle oneself)	24-h Prediction Window
Georga et al. (2013) [33]	Multicenter	T1DM	40.3 ± 13.50	15	CGM, Patients’ Records, Activity Monitor	Prospective study	Hypoglycemia (two consecutive blood glucose values ≤ 3.9 mmol/L and lasting at least 10 min)	30-min and 60-min Prediction Windows
Guemes et al. (2020) [34]	The United States	T1DM	50.0 ± /	6	OhioT1DM Dataset	Retrospective study	Hypoglycemia (CGM <3.9 mmol/L or requiring emergency rescue)	Nighttime
Lebech et al. (2024) [35]	Denmark	T1DM	68.0 ± /	205	CGM	Retrospective study	Hypoglycemia (blood glucose <3.9 mmol/L)	One-week Prediction Window
Mosquera et al. (2023) [36]	The United States	T1DM	38.0 ± 13.00	50	Tidepool Dataset	Retrospective study	Hypoglycemia (blood glucose <3.9 mmol/L)	Daily Activity
Oviedo et al. (2019) [37]	Spain	T1DM	41.0 ± 10.00	10	CGM, insulin Pump	Retrospective study	Hypoglycemia (blood glucose <3.9 mmol/L or insulin pump stopped for >70 min)	4-h Prediction Window after Meal
Oviedo et al. (2019) [38]	Spain	T1DM	41.0 ± 10.00	10	Insulin Pump, CGM	Retrospective study	Hypoglycemia (≤3.9 mmol/L or <3.0 mmol/L)	240-min Prediction Window after Meal/Insulin Bolus
Parcerisas et al. (2022) [39]	Spain	T1DM	31.8 ± 16.80	10	CGM、Body Activity Monitor	Retrospective study	Hypoglycemia (three consecutive blood glucose values below 3.9 mmol/L)	Nighttime
Reddy et al. (2019) [40]	The United States	T1DM	33.0 ± 6.00	55	Clinical Research Data	Retrospective study	Hypoglycemia (capillary blood glucose value < 3.9 mmol/L during or after exercise)	Exercise
Sampath et al. (2016) [41]	Ukraine	T1DM	41.5 ± /	34	Dataset of EU FP7 Project	Retrospective study	Hypoglycemia	Nighttime
Tyler et al. (2022) [42]	The United States	T1DM	34.5 ± 4.70	20	Clinical Exercise Research Dataset	Prospective study	Hypoglycemia (blood glucose <3.9 mmol/L)	Prediction Window during exercise and within 4 h after the start of exercise
Vu et al. (2019) [43]	The United States	T1DM	45.3 ± 16.38	9,800	CGM	Retrospective study	Hypoglycemia (blood glucose <3.9 mmol/L and lasting ≥15 min)	Nighttime

Population: all are adults (≥18 years old); /: relevant data not provided; CGM: continuous glucose monitoring; The conversion relationship between the blood glucose unit “mg/dL” and “mmol/L” in this article is 1 mmol/L = 18 mg/dL. The blood glucose criteria for diagnosing hypoglycemia in this study, 3.9 mmol/L, is equivalent to 70 mg/dL, and 3.0 mmol/L is equivalent to 54 mg/dL.

### 3.3 Methodological quality assessment

The internationally recognized PROBAST tool was employed to assess the risk of bias and clinical applicability of the included studies [24]. As presented in Table 2, among the 18 studies, only one study [27] was deemed to have a low risk of bias, while the remainder exhibited varying levels of methodological deficiencies.

**Table 2:** Risk of bias and applicability assessment of included studies.

Author(s) (Year)	Risk of bias				Applicability			Overall	
	Study subjects	Predictors	Outcome	Analysis	Study subjects	Predictors	Outcome	Risk of bias	Applicability
Shao et al. (2024) [19]	−	+	+	−	+	+	+	−	+
Afentakis et al. (2025) [27]	+	+	+	+	+	+	+	+	+
Bergford et al. (2023) [28]	+	+	+	−	+	+	+	−	+
Berikov et al. (2022) [29]	−	+	+	−	+	+	+	−	+
Bertachi et al. (2020) [30]	+	+	+	−	+	+	+	−	+
Cichosz et al. (2014) [31]	−	+	+	−	+	+	+	−	+
Cox et al. (2007) [32]	+	+	−	?	?	?	?	−	?
Georga et al. (2013) [33]	−	−	−	−	?	?	?	−	?
Guemes et al. (2020) [34]	−	+	−	−	−	+	+	−	−
Lebech et al. (2024) [35]	+	+	+	?	+	+	+	?	+
Mosquera et al. (2023) [36]	−	−	−	−	−	−	−	−	−
Oviedo et al. (2019) [37]	−	−	+	−	−	+	+	−	−
Oviedo et al. (2019) [38]	−	−	−	−	?	?	?	−	?
Parcerisas et al. (2022) [39]	+	+	−	−	+	+	+	−	+
Reddy et al. (2019) [40]	+	+	+	?	+	+	+	?	+
Sampath et al. (2016) [41]	−	−	−	−	−	+	+	−	−
Tyler et al. (2022) [42]	−	−	?	?	?	?	?	−	?
Vu et al. (2020) [43]	−	?	−	−	?	?	?	−	?

“+” Indicates low risk/high applicability, “−” indicates high risk/poor applicability, “?” indicates unclear.

The risk of bias (ROB) assessment covered four key domains. In the study domain, seven studies [27], 28], 30], 32], 35], 39], 40] were assessed as low risk. In contrast, 11 studies (61.1 %) [19], 29], 31], 33], 34], [36], [37], [38, [41], [42], [43] were at high risk, primarily due to retrospective designs, small sample sizes, or reliance on single data sources. Moving to the predictor domain, 11 studies [19], [27], [28], [29], [30], [31], [32, 34], 35], 39], 40] were categorized as low risk, while six studies (33.3 %) [33], [36], [37], [38, 41], 42] were high risk and one study [43] had an unclear risk status. High risk in this domain was mainly due to overlapping predictors with outcome definitions or inconsistencies in predictor measurement.

For the outcome domain, nine studies [19], [27], [28], [29], [30], [31, 35], 37], 40] were assessed as low risk, whereas eight studies (44.4 %) [32], [33], [34, 36], 38], 39], 41], 43] were high risk, primarily due to reliance on predictor data or poorly defined outcomes. One study [42] had an unclear risk in this domain.

The analysis domain showed the most pronounced risk patterns, with only one study [27] classified as low risk and 13 studies (72.2 %) [19], [28], [29], [30], [31, 33], 34], [36], [37], [38], [39, 41], 43] as high risk. High risk in the analysis domain was primarily linked to incomplete handling of missing data, univariate screening of predictors, failure to address overfitting, and limited sample sizes, with four studies [32], 35], 40], 42] having an unclear risk status.

The applicability assessment focused on three core aspects. Regarding participants, nine studies [19], [27], [28], [29], [30], [31, 35], 39], 40] demonstrated high applicability, while the remaining nine studies exhibited low or unclear applicability due to participant populations not fully representative of real-world clinical settings. For predictors, 12 studies [19], [27], [28], [29], [30], [31, 34], 35], 37], [39], [40], [41] showed high applicability, whereas six studies had low or unclear applicability, largely due to reliance on historical data for predictor measurement. In terms of outcomes, 12 studies [19], [27], [28], [29], [30], [31, 34], 35], 37], [39], [40], [41] were assessed as high applicability, while six studies had low or unclear applicability resulting from inconsistencies in outcome definitions and clinical criteria.

Overall, only one study [27], representing 5.6 % of all included studies, met both the low-risk-of-bias and high-applicability criteria. The remaining studies exhibited varying degrees of methodological limitations that may impact the reliability and generalizability of their findings.

### 3.4 Model construction features

#### 3.4.1 Predictors and modeling methods

A total of 44 modeling algorithms were adopted across the included studies. Machine learning algorithms accounted for 87.5 % (*n* = 28), among which random forest (*n* = 8) [19], [27], [28], [29, 34], 36], 40], 43] and support vector machine (*n* = 8) [19], 27], 30], 33], 34], [37], [38], [39] were the most frequently applied approaches. Traditional regression models represented only 12.5 % (*n* = 4) [29], 31], 34], 42]. Machine learning algorithms demonstrated notable advantages in scenarios involving multimodal data integration, owing to their capacity to process high-dimensional data and identify complex nonlinear relationships [27], 29]. Notably, these machine learning models typically incorporate all available variables as inputs, without performing traditional multivariate regression analysis beforehand. Consequently, there is often no formal variable screening process, limiting the precise assessment of the independent effect size and statistical significance of individual predictors.

The predictors themselves exhibited considerable heterogeneity. Core variables included dynamic glycemic characteristics such as the coefficient of variation in blood glucose and the time spent below target range from CGM data, insulin-related metrics like basal insulin dose and residual active insulin, clinical features including diabetes duration, glycosylated hemoglobin levels, and body mass index, and behavioral parameters such as exercise intensity and carbohydrate intake.

#### 3.4.2 Model validation and presentation

There was substantial variation in the model validation strategies. Internal validation was predominant, used in 17 studies (94.4 %) [19], [27], [28], [29], [30], [31], [32], [33], [34], [35], [36], [37], [38], [39], [40, 42], 43], with 10-fold cross-validation being the most common method [19], 27], 30], 31], 35], [38], [39], [40, 43], followed by the leave-one-out method [29], 30], 33], 36]. Only eight studies conducted external validation [19], 27], 30], 35], 36], [38], [39], [40]. Of these, five employed independent cohort validation [27], 30], 35], 36], 38], and two studies [28], 33] performed cross-center validation. Rigorous external validation is critical for evaluating the generalizability of these models.

Most models were presented as mathematical formulas or integrated into digital systems. Six studies [27], 32], 34], [40], [41], [42] provided mathematical formula representations. Four studies [32], 37], 38], 43] integrated prediction models into e-health systems. Notably, only a web-based calculator tool was developed [36], that uses four basic parameters to generate exercise-related hypoglycemia risk scores, significantly enhancing the clinical usability of their model.

However, several methodological limitations should be acknowledged. Eleven studies [19], 28], 29], [32], [33], [34, 37], 39], [41], [42], [43] did not clearly report how the validation set was divided, and seven studies [19], 28], 29], 32], 39], 41], 43] failed to specify the chronological sequence of the training and validation sets, potentially introducing temporal bias. Furthermore, four studies [28], 31], 37], 38] employed a retrospective validation design, in which the validation cohort was essentially a temporal subset of the original dataset, thereby undermining the robustness of the validation findings. The core indicators of the models included in this analysis are summarized in Table 3.

**Table 3:** Algorithmic features and performance metrics of hypoglycemia risk prediction models in adults with type 1 diabetes.

Author(s) (Year)	Modeling method	Core predictors	Core indicators of prediction performance	Validation method	Presentation form
Shao et al. (2024) [19]	SVM/RF/ LSTM	Dynamic features of CGM + clinical indicators	LSTM: AUC 0.96 (severe hypoglycemia) 0.93 (mild hypoglycemia) SVM: AUC 0.94 (severe hypoglycemia) 0.90 (mild hypoglycemia) RF: AUC 0.95 (severe hypoglycemia) 0.90 (mild hypoglycemia)	Hold-out method + external validation	Not specified
Afentakis et al. (2025) [27]	SVM/RF	Dynamic features of CGM	SVM: AUC 0.79 RF: AUC 0.76	10-Fold cross-validation + external validation	Mathematical formulas
Bergford et al. (2023) [28]	RMRF/RMLR	Exercise parameters + CGM + insulin dose	RMRF: AUC 0.83 RMLR: AUC 0.83	Hold-out method	Not specified
Berikov et al. (2022) [29]	RF/ANN/ Log R Lasso	Dynamic features of CGM + clinical indicators	RF: AUC 0.97 (15 min) 0.94 (30 min) Log R Lasso: AUC 0.97 (15 min) 0.94 (30 min) ANN: AUC 0.95 (15 min) 0.92 (30 min)	10-Fold cross-validation	Not specified
Bertachi et al. (2020) [30]	MLP/SVM	CGM + exercise data	MLP: comprehensive index 0.78 SVM: comprehensive index 0.80	5-Fold cross-validation	Not specified
Cichosz et al. (2014) [31]	Logistic	CGM + HRV	AUC 0.98 (sensitivity 79 %, specificity 99 %)	Hold-out method	Not specified
Cox et al. (2007) [32]	SA	Blood glucose fluctuations + insulin dose	Accuracy 63 %	Prospective internal validation	Mathematical formulas/system integration
Georga et al. (2013) [33]	SVM	CGM + activity data	Nighttime: sensitivity 94 % Daytime: sensitivity 92 %	10-Fold cross-validation	Not specified
Guemes et al. (2020) [34]	RF/ANN/SVM/Logistic	Nocturnal blood glucose features	RF: AUC 0.72 ANN: AUC 0.69 SVM: AUC 0.67 logistic: AUC 0.65	10-Fold cross-validation	Mathematical formulas
Lebech et al. (2024) [35]	XGBoost	CGM + clinical indicators + HRV	Model 1: AUC 0.87 Model 2: AUC 0.83	Hold-out method + external validation	Not specified
Mosquera et al. (2023) [36]	RF	Four exercise-related factors	1-h prediction: AUC 0.83 24-h prediction: AUC 0.66	3-Fold cross-validation + external validation	Web calculator
Oviedo et al. (2019) [37]	NB/AB/SVM/ANN	Postprandial blood glucose + insulin infusion	Blood glucose < 70 mg/dL: Average sensitivity 0.49, average specificity: 0.74 Blood glucose <54 mg/dL: Average sensitivity 0.51, average specificity: 0.74	5-Fold cross-validation	Medical record system integration
Oviedo et al. (2019) [38]	SVM	Postprandial blood glucose fluctuations + insulin dose	Level 1: specificity 79 %, sensitivity 71 % Level 2: specificity 81 %, sensitivity 77 %	5-Fold cross-validation + external validation	System integration
Parcerisas et al. (2022) [39]	SVM	Nocturnal features of CGM	AUC 0.81 (sensitivity 73 %, Specificity 72 %)	5-Fold cross-validation + external validation	Not specified
Reddy et al. (2019) [40]	DT/RF	Blood glucose at exercise onset + residual insulin amount	DT: accuracy 0.79 RF: accuracy 0.97	10-Fold cross-validation + external validation	Graphic scoring table/mathematical formulas
Sampath et al. (2016) [41]	LFSRR	Blood glucose control index	Sensitivity 80 %, specificity 96 %, F1-score 84 %	External validation	Mathematical formulas
Tyler et al. (2022) [42]	Logistic	Exercise-related factors	Sensitivity 64 %, accuracy 61 %	Hold-out method	Mathematical formulas
Vu et al. (2019) [43]	RF	Nocturnal CGM data	Overall night: AUC 0.84 Early night (0–3 am): AUC 0.90 Late night (3–6 am): AUC 0.75	10-Fold cross-validation	Medical record system integration

Abbreviations of algorithms: SVM, support vector machine; RF, random forest; DT, decision tree; MLP, multilayer perceptron; XGBoost, extreme gradient boosting; HRV, heart rate variability; LSTM, long short-term memory network; ANN, artificial neural network; RMLR, repeated measures logistic regression; RMRF, repeated measures random forest; LFSRR, linear functional strategy ranking algorithm; SA, sliding algorithm; NB, Gaussian Naive Bayes; AB, adaptive boosting algorithm; Log R Lasso, logistic regression with L1 regularization; “**+**” represents external validation; AUC, area under the receiver operating characteristic curve, reflecting the overall discriminative ability of the model; Comprehensive index: A synthetic indicator for evaluating overall model performance; Accuracy: the proportion of all correctly classified cases among total cases; Sensitivity: The proportion of true positive cases correctly identified by the model; Specificity: The proportion of true negative cases correctly identified by the model; F1-score: The harmonic mean of precision and sensitivity, balancing model classification performance.

### 3.5 Model performance analysis

#### 3.5.1 Model performance characteristics

Among the 42 prediction models developed across the included studies, 29 reported the AUC values with an average of 0.85. A total of 15 high-performing models (AUC ≥ 0.85) were identified. These high-performing models shared a common feature: the integration of multimodal data fusion techniques. The median sensitivity and specificity were 75 % (IQR 64–80 %) and 79 % (IQR 74–96 %), respectively. The most balanced performance was observed in the logistic regression model [31], which achieved a sensitivity of 79 %, specificity of 99 %, and a Youden index of 0.78. This makes it particularly suitable for clinical screening scenarios where high specificity is critical. Interestingly, three studies [27], 36], 40] reported on exercise-specific models, with AUC values ranging from 0.76 to 0.83. These were slightly lower than those of nighttime prediction models (AUC 0.84–0.97), likely reflecting the more complex blood glucose fluctuations associated with exercise.

Methodological shortcomings were also noted. Eleven studies did not fully report performance indicators. Of these, five did not provide specific data [19], 34], 35], 37], 43], three did not report sensitivity [28], 33], 41], and two did not specify optimal cut-off values [30], 39]. Furthermore, six studies [19], 27], 29], 35], 36], 43] utilized non-standard performance metrics, such as composite measures, reducing the comparability of their findings.

In terms of clinical translation value, prediction models from five studies [32], [36], [37], [38, 43] implemented via web calculators, system integration, or electronic medical record system integration demonstrated direct clinical applicability and could be directly translated into clinical practice. In contrast, models from 13 studies [19], [27], [28], [29], [30], [31, [33], [34], [35, [39], [40], [41], [42] that only provided mathematical formulas, graphic scoring tables, or lacked detailed implementation information remained research tools, which require further development and integration prior to clinical application.

#### 3.5.2 Meta-analysis of AUC values

**Figure 2:**
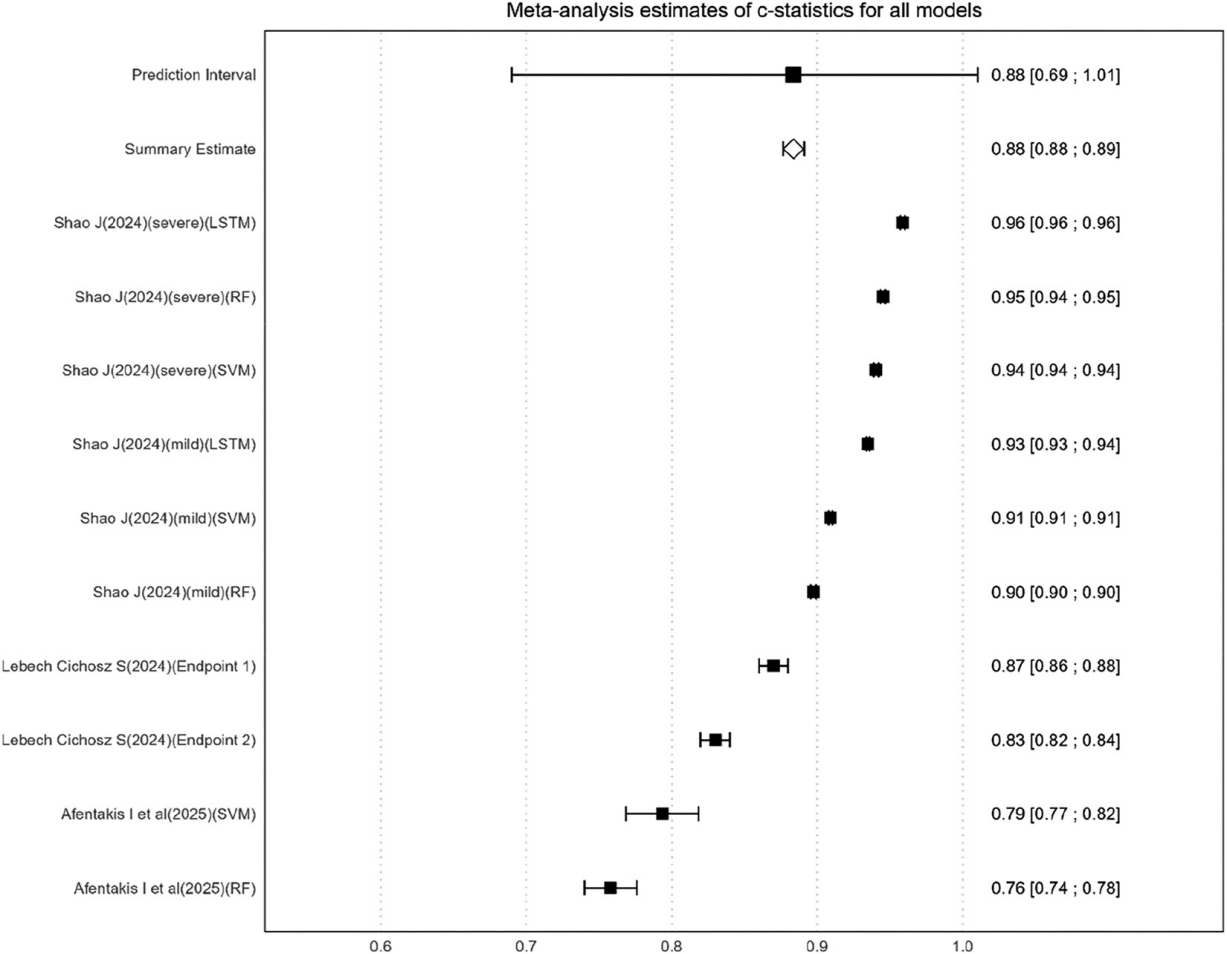
Forest plot of meta-analysis for pooled AUC of hypoglycemia prediction models.

A meta-analysis of 10 models from three studies [19], 27], 35] (Figure 2) revealed a pooled AUC of 0.88 (95 % CI: 0.88–0.89) using a random-effects model. These confidence intervals are well above the threshold for clinical failure (AUC = 0.5), demonstrating statistically significant predictive ability.

There was considerable substantial between-study heterogeneity (*I* ^2^ = 99.82 %, 95 % CI: 99.79–99.84), and Cochran’s *Q* test confirmed the presence of significant heterogeneity (*Q* = 5,009.90, d*f* = 9, *P* < 0.001). Notably, the absolute heterogeneity measure (*τ* ^2^ = 0.00056), indicating that the observed variation in effect sizes across studies was largely attributable to measurement error rather than genuine clinical heterogeneity. High heterogeneity suggests that the pooled predictive performance may not be directly generalizable across different clinical settings, and caution is warranted when applying the pooled AUC in practice. Although subgroup analyses were performed based on prediction time windows (nighttime, exercise, postprandial), data types (CGM/clinical records), and modeling algorithms, heterogeneity remained high. The observed heterogeneity may also stem from variability in study design and diverse prediction time windows. To provide a more conservative estimate of the pooled effect size, a random-effects model was used, indicating that the clinical generalizability of the predictive performance of existing models should be interpreted with caution. The Egger test (*t* = −1.365, *P* = 0.209) showed no statistically significant publication bias.

Despite the substantial heterogeneity, the findings of this study still provide important reference for clinical practice. The pooled AUC of 0.888 indicated that current models have moderate predictive ability for hypoglycemia in adults with T1DM, with relatively better performance observed in the nighttime setting. It is recommended that externally validated models should be prioritized for clinical use, with dynamically adjustments based on individual patient characteristics [19].

## 4 Discussion

### 4.1 Predictive efficacy and methodological challenges of hypoglycemia risk prediction models in adults with T1DM

#### 4.1.1 Predictive performance of the models

The 18 prediction models included in this analysis demonstrated an overall average AUC of 0.85, indicating moderate-to-good performance in predicting hypoglycemic events. The random forest model developed by Berikov [29] using multimodal data achieved the highest performance with an AUC of 0.97 for the 15-min prediction window. Meta-analysis confirmed a pooled AUC of 0.888 (95 % CI: 0.840–0.936), although substantial between-study heterogeneity was observed (*τ* ^2^ = 0.00056). This pooled effect size supports the general predictive ability of current models, and the subgroup findings further highlight superior performance in the nighttime prediction scenario. These quantitative results directly reinforce the need to prioritize robust, well-validated models for clinical use, especially for nocturnal hypoglycemia warning.

Importantly, model performance was strongly associated with the type of input data. Models using dynamic CGM-derived indicators achieved a higher mean AUC of 0.89 than those relying only on static clinical indicators of 0.70, underscoring the value of real-time monitoring data and providing data support for clinical real-time early warning. The simplified model showed that a parsimonious four-predictors set still yielded an AUC of 0.83, suggesting that streamlined models may improve clinical applicability without substantial loss of accuracy and address the pain point of difficult implementation of complex models in clinical practice [36]. These findings support the core observation of this study, which highlights the good predictive performance of existing models, particularly those incorporating dynamic CGM data.

#### 4.1.2 Methodological limitations of existing models

The PROBAST assessment identified critical methodological limitations. Overall, 88.9 % of studies had high or unclear risk of bias, mainly due to the retrospective design of 61.1 % of studies, which often introduced temporal mismatch between predictors and outcomes and reduced the accuracy of model prediction and the ability of causal inference [19]. Many studies did not report strategies for missing data, potentially introducing selection bias [30] Additionally, many machine learning models used all variable input without feature selection, limiting clinical interpretability [27].

Only 44.4 % of the included studies conducted external validation, which included 18 models. The other 24 models, accounting for 57.14 % of all included models, were not externally validated. This high proportion of models without external validation severely restricts the generalisability of the study findings, as unvalidated models may not perform consistently when applied to different clinical populations or settings, thereby highlighting an important research gap in the field.

Despite recent advances in predictive performance, the methodological shortcomings of existing models limit their effective clinical implementation. Clinically, externally validated models, such as the LSTM model by Shao et al. (AUC = 0.959) [19], should be prioritized and integrated with CGM data to provide accurate early warnings. For high-risk patients with autonomic neuropathy, intensified nighttime blood glucose monitoring and timely updates to the prediction model are essential to improve preventive intervention accuracy.

### 4.2 Developmental characteristics and technical bottlenecks of hypoglycemia risk prediction models in adults with T1DM

#### 4.2.1 Characteristics of the primary stage of evidence generation

Among the 18 studies reviewed in this research, 11 studies [19], 29], [34], [35], [36], [37], [38], [39], [40], [41, 43] were single-center retrospective studies with a median sample size of just 34 cases. Some of these studies did not include external validation, highlighting that current model development is largely exploratory and lacks a robust foundation for clinical translation. Notably, only six studies [28], [30], [31], [32], [33, 42] employed prospective study designs, providing stronger causal evidence for model development through a sound chronological structure.

The diversity of predictors across studies underscores this exploratory nature. The number of variables incorporated varied widely, encompassing factors such as diabetes duration, dynamic CGM-derived measures such as blood glucose coefficient of variation, insulin dosages, and exercise parameters. Machine learning models dominated, yet only three studies [32], 36], 40] reported factor weights, limiting the interpretability of these models. Lebech Cichosz [35] included 26 predictors in their XGBoost model with an AUC of 0.87. In contrast, Mosquera-Lopez [36] achieved an AUC of 0.83 using only four basic factors, illustrating that streamlined predictor sets can enhance model feasibility in clinical practice.

A polarized trend in modeling approaches emerged. High-dimensional machine learning methods including random forests and support vector machines were used in 89 % of studies. Afentakis [27] integrated 10 CGM features to build a model with an AUC of 0.79. However, the black-box nature of these machine learning models presents challenges for clinical interpretation. In contrast, 11 % of studies relied on traditional statistical methods such as logistic regression, exemplified by Cox et al.; while their model, based on three clinical indicators, achieved a lower predictive accuracy of 63 %, it offered high interpretability and produced a key advantage for clinical decision-making.

#### 4.2.2 Analysis of practical barriers to clinical translation

This review revealed several barriers that limit the clinical applicability of these prediction models. Only three studies [32], 37], 43] explicitly reported integrating their models into e-health systems. Prediction time windows tended to focus on nighttime or exercise scenarios, limiting applicability to daily life. Moreover, most studies did not discuss mechanisms for model updates. Considering the dynamic metabolic state of patients with T1DM, a lack of model adaptation over time can compromise prediction accuracy. It is recommended to cooperate with medical institutions and technology enterprises to embed multi-center validated models into existing CGM devices or hospital information systems, realizing real-time linkage between early warning information and clinical intervention.

### 4.3 Challenges and coping strategies in addressing global population heterogeneity for the generalization of hypoglycemia prediction models

#### 4.3.1 Significant effects of ethnicity-specific metabolic signatures on model performance

Significant population heterogeneity affects the generalizability of prediction models. Currently, the development of hypoglycemia prediction models predominantly relies on cohorts from European and American populations, which represent 83 % of the included studies. Considerable metabolic differences exist across T1DM patients from diverse ethnic backgrounds. The decline rate of β-cell function is higher in Asian populations than in Caribbean populations, and insulin sensitivity is also affected by genetic background and dietary habits [44]. In this study, it was observed that the AUC value of the LSTM model decreased by an average of 3 % when validation in Asian cohorts compared with corresponding models in European and American populations [19]. This observation supports the substantial influence of ethnicity-specific metabolic features on model performance. The LADA subtype accounts for up to 6.1 % of the Asian T1DM population and presents a distinct islet autoantibody profile compared with classical T1DM [45]. Unfortunately, existing models have not performed subgroup analyses in this population, which may lead to biased predictive outcomes.

#### 4.3.2 Constraints on clinical implementation due to limited access to dynamic monitoring technologies

The integration of CGM data substantially improves the performance of hypoglycemia prediction models, but widespread real-world clinical implementation is severely limited by high device costs and recurring service expenses. These economic constraints are particularly burdensome in low and middle-income countries, where health resources and CGM coverage are severely restricted. Yet, globally, only 25 % of T1DM patients have access to CGM. Many facilities in low-income countries still depend on capillary glucose testing, which restricts the data dimension available for model perdiction [46]. The simplified model [36], which uses only four basic parameters, achieves an AUC of 0.83. Standardized collection of routine indicators may partly alleviate the technology gap in resource-limited regions. However, model calibration decreases by 15 % in cohorts without CGM support. Overreliance on costly monitoring equipment creates a substantial barrier to real-world implementation across regions with unequal health resources. These economic and accessibility differences should be explicitly addressed to achieve equitable global application of prediction models, especially for underserved populations in low-income settings with limited CGM access.

#### 4.3.3 Systematic research gaps in predictive modeling for special populations

Existing studies provide insufficient attention to special populations such as pregnant women with T1DM and individuals with brittle diabetes [47], 48]. Pregnancy women account for 12 % of adult T1DM cases. The predictive accuracy reported by Georga [33] decreased by 22 % in pregnancy cohorts, reflecting unique metabolic patterns during pregnancy. Elderly T1DM patients (>65 years) often present multiple comorbidities and large variations in insulin sensitivity. These factors lead to poor model calibration generally below 0.75. Although children and adolescents with T1DM were excluded from this study, previous evidence indicates a 36 % higher nocturnal hypoglycemia incidence in younger patients than in adults [49]. Age-specific prediction models are therefore urgently needed.

### 4.4 Model interpretability and ethical challenges: a double-edged sword for clinical translation

#### 4.4.1 Explainability and clinical acceptance: overcoming the “black box” dilemma of machine learning

Although machine learning models often outperform traditional approaches in predictive performance, their inherent “black box” nature poses a significant challenge for clinical translation [50]. This study found that only a few models attempted to enhance interpretability through feature importance analysis; most failed to clarify the clinical significance of individual predictors. Berikov’s random forest model achieved an impressive AUC of 0.97, yet relied on 26 CGM-derived indicators, leaving clinicians unable to understand how measures like the coefficient of variation (CV) of blood glucose specifically contribute to hypoglycemia risk [29]. This issue is particularly pronounced in emergency scenarios, where even high-sensitivity models may be used cautiously due to the absence of clear, intuitive explanations [51].

Alignment between prediction models and T1DM clinical guidelines is critical for successful translation. Current international clinical guidelines for T1DM emphasize consistent glucose monitoring, individualized insulin dosing, and early identification of hypoglycemia risk factors [52]. However, most existing prediction models focus primarily on short-term hypoglycemia forecasting rather than integrating guideline-recommended targets for glucose range or hypoglycemia prevention strategies. Additionally, many models lack formal validation against guideline defined risk thresholds for hypoglycemia. This mismatch between model design and clinical guideline requirements may limit acceptance among clinicians and reduce the models’ real-world utility. Future models should be designed to support rather than replace guideline directed care by incorporating evidence-based risk factors and generating outputs that align with standard clinical decision pathways.

#### 4.4.2 Data privacy and ethical governance: hidden barriers to global model deployment

This review also highlights an overlooked challenge of data privacy and ethical governance. Most current models rely heavily on CGM and smart device data, yet few studies explicitly address data anonymization or privacy protection mechanisms. Vu [43] developed a model using CGM data from 9,800 patients but did not detail how they mitigated the risk of patient re-identification. These issues become more complex in cross-regional collaborations. In the Asian cohort study by Shao [19], data-sharing restrictions impeded fair generalization assessments. Moreover, patients in low-income regions may be excluded from the benefits of these models due to disparities in technology access, such as limited CGM use, exacerbating existing health inequities [53].

### 4.5 Study limitations and future perspectives

This review has several limitations that should be acknowledged. First, the included studies were predominantly single-center retrospective designs, which may introduce selection bias and limit the generalizability of the findings. Second, most models lacked external validation, raising concerns about their consistency in different clinical settings. Third, the majority of studies were based on European and American cohorts, failing to adequately represent the metabolic heterogeneity of T1DM patients from diverse ethnic backgrounds, particularly Asian populations and special subgroups such as LADA, pregnant women, and the elderly. Additionally, the overreliance on CGM data, which is not universally accessible, limits the real-world applicability of many high-performance models. Finally, few studies addressed model interpretability and data privacy, which are critical for clinical acceptance and ethical deployment.

Future studies should focus on Strengthening prospective cohort designs and standardizing data processing. Enhancing model reliability through multi-center external validation. Incorporating clinically relevant variables using expert consensus or rule-based systems during machine learning model development. Employing regularization techniques to balance model complexity and predictive power. Ensuring consistency between domain knowledge and data-driven features, minimizing over-reliance on subjective experience. In clinical practice, multi-center validated models, particularly those incorporating dynamic CGM data, should be favored. Monitoring frequency should be personalized based on patient-specific factors. Furthermore, implementing a closed-loop feedback mechanism after interventions can verify early-warning accuracy, ensuring a balance between model performance and clinical applicability. Additionally, future research should prioritize addressing global population heterogeneity, developing low-cost, accessible models for resource-limited settings, enhancing model interpretability through explainable AI tools, and establishing standardized ethical and data governance protocols to promote equitable global application.

## 5 Conclusions

This study represents the first comprehensive synthesis of hypoglycemia risk prediction models in adults with T1DM. Machine learning approaches show clear benefits in integrating multimodal data, and CGM-derived indicators are essential for improving predictive performance. Overall, these models demonstrate strong and reliable predictive capacity, with high-performing models achieving clinically useful accuracy. However, high heterogeneity, methodological limitations, and insufficient external validation restrict the clinical translation and generalizability of current models.

Notably, this research proposes an “evidence-driven risk management pathway,” that integrates patient-specific early warning thresholds, real-time wearable device integration, and continuous glucose monitoring for targeted interventions. Multi-center validated models should be prioritized in clinical practice. These findings strengthen the evidence-based for optimizing diabetes management and translating predictive models into clinical decision-making.

To improve global applicability, future predictive models should explicitly include elderly individuals, pregnant patients, and those with latent autoimmune diabetes in adults. Meanwhile, unequal access to continuous glucose monitoring in low-resource settings represents a key practical barrier that should be addressed to ensure equitable public health benefits. Future work should focus on standardized data governance, interpretable artificial intelligence, and population-specific models to build a comprehensive lifelong prevention system.

## Supplementary Material

Supplementary Material

Supplementary Material

## References

[1] Ishtiak-Ahmed K, Carstensen B, Pedersen-Bjergaard U, Jørgensen ME (2017). Incidence trends and predictors of hospitalization for hypoglycemia in 17,230 adult patients with type 1 diabetes: a Danish register linkage cohort study. Diabetes Care.

[2] Zhang Q, Zhou H, Lin R, Hu L, Zhu X (2025). Risk factors for hypoglycaemia in adults with type 1 diabetes: a systematic review and meta-analysis. BMC Endocr Disord.

[3] Khunti K, Alsifri S, Aronson R, Cigrovski Berković M, Enters-Weijnen C, Forsén T (2016). Rates and predictors of hypoglycaemia in 27 585 people from 24 countries with insulin-treated type 1 and type 2 diabetes: the global HAT study. Diabetes Obes Metab.

[4] Seaquist ER, Anderson J, Childs B, Cryer P, Dagogo-Jack S, Fish L (2013). Hypoglycemia and diabetes: a report of a workgroup of the American diabetes association and the endocrine society. Diabetes Care.

[5] Emral R, Pathan F, Cortés CAY, El-Hefnawy MHF, Goh SY, Gómez AM (2017). Self-reported hypoglycemia in insulin-treated patients with diabetes: results from an international survey on 7289 patients from nine countries. Diabetes Res Clin Pract.

[6] Ali NH, Al-Kuraishy HM, Al-Gareeb AI, Hadi NR, Assiri AA, Alrouji M (2024). Hypoglycemia and Alzheimer disease risk: the possible role of dasiglucagon. Cell Mol Neurobiol.

[7] Christou MA, Christou PA, Kyriakopoulos C, Christou GA, Tigas S (2023). Effects of hypoglycemia on cardiovascular function in patients with diabetes. Int J Mol Sci.

[8] Abdel-latif R, Heeba GH, Hassanin SO, Waz S, Amin A (2022). TLRs-JNK/NF-κB pathway underlies the protective effect of the sulfide salt against liver toxicity. Front Pharmacol.

[9] Gui W, Zhu WF, Zhu Y, Tang S, Zheng F, Yin X (2020). LncRNAH19 improves insulin resistance in skeletal muscle by regulating heterogeneous nuclear ribonucleoprotein A1. Cell Commun Signaling.

[10] Yang W, Lyu Y, Xiang R, Yang J (2022). Long noncoding RNAs in the pathogenesis of insulin resistance. Int J Mol Sci.

[11] Amin A, Lotfy M, Mahmoud-Ghoneim D, Adeghate E, Al-Akhras MA, Al-Saadi M (2011). Pancreas-protective effects of chlorella in STZ-induced diabetic animal model: insights into the mechanism. J Diabetes Mellit.

[12] Hamza AA, Fikry EM, Abdallah W, Amin A (2018). Mechanistic insights into the augmented effect of bone marrow mesenchymal stem cells and thiazolidinediones in streptozotocin-nicotinamide induced diabetic rats. Sci Rep.

[13] Al Shamsi MS, Amin A, Adeghate E (2004). Beneficial effect of vitamin E on the metabolic parameters of diabetic rats. Mol Cell Biochem.

[14] Al Shamsi M, Amin A, Adeghate E (2006). Vitamin E ameliorates some biochemical parameters in normal and diabetic rats. Ann N Y Acad Sci.

[15] Frier BM (2014). Hypoglycaemia in diabetes mellitus: epidemiology and clinical implications. Nat Rev Endocrinol.

[16] Kaur J, Seaquist ER (2023). Hypoglycaemia in type 1 diabetes mellitus: risks and practical prevention strategies. Nat Rev Endocrinol.

[17] Nomura A, Noguchi M, Kometan M, Furukawa K, Yoneda T (2021). Artificial intelligence in current diabetes management and prediction. Curr Diabetes Rep.

[18] Acuna E, Aparicio R, Palomino V (2023). Analyzing the performance of transformers for the prediction of the blood glucose level considering imputation and smoothing. Big DATA Cogn Comput.

[19] Shao J, Pan Y, Kou WB, Feng H, Zhao Y, Zhou K (2024). Generalization of a deep learning model for continuous glucose monitoring–based hypoglycemia prediction: algorithm development and validation study. JMIR Med Inf.

[20] Liu C, Huang Z, Liu T, Ge Y, Yuan J, Lin Y (2025). Construction and validation of a hypoglycemia risk prediction model for hospitalized type 2 diabetes patients based on machine learning. BMC Endocr Disord.

[21] Ellahham S (2020). Artificial intelligence: the future for diabetes care. Am J Med.

[22] Moons KG, Hooft L, Williams K, Hayden JA, Damen JA, Riley RD (2018). Implementing systematic reviews of prognosis studies in cochrane. Cochrane Database Syst Rev.

[23] Moher D, Liberati A, Tetzlaff J, Altman DG, The Prisma Group (2009). Preferred reporting items for systematic reviews and meta-analyses: the PRISMA statement. PLoS Med.

[24] Moons KGM, Wolff RF, Riley RD, Whiting PF, Westwood M, Collins GS (2019). PROBAST: a tool to assess risk of bias and applicability of prediction model studies: explanation and elaboration. Ann Intern Med.

[25] Fernandez-Felix BM, López-Alcalde J, Roqué M, Muriel A, Zamora J (2023). CHARMS and PROBAST at your fingertips: a template for data extraction and risk of bias assessment in systematic reviews of predictive models. BMC Med Res Methodol.

[26] de Jong Y, Ramspek CL, Zoccali C, Jager KJ, Dekker FW, van Diepen M (2021). Appraising prediction research: a guide and meta-review on bias and applicability assessment using the prediction model risk of bias ASsessment tool (PROBAST). Nephrol (Carlt Vic,).

[27] Afentakis I, Unsworth R, Herrero P, Oliver N, Reddy M, Georgiou P (2025). Development and validation of binary classifiers to predict nocturnal hypoglycemia in adults with type 1 diabetes. J Diabetes Sci Technol.

[28] Bergford S, Riddell MC, Jacobs PG, Li Z, Gal RL, Clements MA (2023). The type 1 diabetes and EXercise initiative: predicting hypoglycemia risk during exercise for participants with type 1 diabetes using repeated measures random forest. Diabetes Technol Ther.

[29] Berikov VB, Kutnenko OA, Semenova JF, Klimontov VV (2022). Machine learning models for nocturnal hypoglycemia prediction in hospitalized patients with type 1 diabetes. J Pers Med.

[30] Bertachi A, Viñals C, Biagi L, Contreras I, Vehí J, Conget I (2020). Prediction of nocturnal hypoglycemia in adults with type 1 diabetes under multiple daily injections using continuous glucose monitoring and physical activity monitor. Sens (Basel Switz).

[31] Cichosz SL, Frystyk J, Hejlesen OK, Tarnow L, Fleischer J (2014). A novel algorithm for prediction and detection of hypoglycemia based on continuous glucose monitoring and heart rate variability in patients with type 1 diabetes. J Diabetes Sci Technol.

[32] Cox DJ, Gonder-Frederick L, Ritterband L, Clarke W, Kovatchev BP (2007). Prediction of severe hypoglycemia. Diabetes Care.

[33] Georga EI, Protopappas VC, Ardigò D, Polyzos D, Fotiadis DI (2013). A glucose model based on support vector regression for the prediction of hypoglycemic events under free-living conditions. Diabetes Technol Ther.

[34] Guemes G, Cappon C, Hernandez H, Reddy R, Oliver O, Georgiou G (2020). Predicting quality of overnight glycaemic control in type 1 diabetes using binary classifiers. IEEE J Biomed Health Inform.

[35] Lebech Cichosz S, Hasselstrøm Jensen M, Schou Olesen S (2024). Development and validation of a machine learning model to predict weekly risk of hypoglycemia in patients with type 1 diabetes based on continuous glucose monitoring. Diabetes Technol Ther.

[36] Mosquera-Lopez C, Ramsey KL, Roquemen-Echeverri V, Jacobs PG (2023). Modeling risk of hypoglycemia during and following physical activity in people with type 1 diabetes using explainable mixed-effects machine learning. Comput Biol Med.

[37] Oviedo S, Contreras I, Bertachi A, Quirós C, Giménez M, Conget I (2019). Minimizing postprandial hypoglycemia in type 1 diabetes patients using multiple insulin injections and capillary blood glucose self-monitoring with machine learning techniques. Comput Methods Progr Biomed.

[38] Oviedo S, Contreras I, Quirós C, Giménez M, Conget I, Vehi J (2019). Risk-based postprandial hypoglycemia forecasting using supervised learning. Int J Med Inf.

[39] Parcerisas A, Contreras I, Delecourt A, Bertachi A, Beneyto A, Conget I (2022). A machine learning approach to minimize nocturnal hypoglycemic events in type 1 diabetic patients under multiple doses of insulin. Sens(Basel Switz).

[40] Reddy R, Resalat N, Wilson LM, Castle JR, El Youssef J, Jacobs PG (2019). Prediction of hypoglycemia during aerobic exercise in adults with type 1 diabetes. J Diabetes Sci Technol.

[41] Sampath S, Tkachenko P, Renard E, Pereverzev SV (2016). Glycemic control indices and their aggregation in the prediction of nocturnal hypoglycemia from intermittent blood glucose measurements. J Diabetes Sci Technol.

[42] Tyler NS, Mosquera-Lopez C, Young GM, El Youssef J, Castle JR, Jacobs PG (2022). Quantifying the impact of physical activity on future glucose trends using machine learning. iScience.

[43] Vu L, Kefayati S, Idé T, Pavuluri V, Jackson G, Latts L (2020). Predicting nocturnal hypoglycemia from continuous glucose monitoring data with extended prediction horizon. AMIA Annu Symp Proc AMIA Symp.

[44] Lee H, Choi J, Kim JI, Watanabe RM, Cho NH, Park KS (2024). Higher genetic risk for type 2 diabetes is associated with a faster decline of β-cell function in an east Asian population. Diabetes Care.

[45] Guglielmi C, Palermo A, Pozzilli P (2012). Latent autoimmune diabetes in the adults (LADA) in Asia: from pathogenesis and epidemiology to therapy. Diabetes Metab Res Rev.

[46] Friedman JG, Cardona Matos Z, Szmuilowicz ED, Aleppo G (2023). Use of continuous glucose monitors to manage type 1 diabetes mellitus: progress, challenges, and recommendations. Pharmacogenomics Pers Med.

[47] Feldman AZ, Brown FM (2016). Management of type 1 diabetes in pregnancy. Curr Diabetes Rep.

[48] Vargas R, Repke JT, Ural SH (2010). Type 1 diabetes mellitus and pregnancy. Rev Obstet Gynecol.

[49] Brunton SA (2007). Nocturnal hypoglycemia: answering the challenge with long-acting insulin analogs. MedGenMed : Medscape Gen Med.

[50] Murdoch WJ, Singh C, Kumbier K, Abbasi-Asl R, Yu B (2019). Definitions, methods, and applications in interpretable machine learning. Proc Natl Acad Sci U S A.

[51] Zhu J, Miao S, Ying R, Li P (2025). Towards unveiling sensitive and decisive patterns in explainable AI with a case study in geometric deep learning. Nat Mach Intell.

[52] Holt RIG, DeVries JH, Hess-Fischl A, Hirsch IB, Kirkman MS, Klupa T (2021). The management of type 1 diabetes in adults. A consensus report by the American diabetes association (ADA) and the european association for the study of diabetes (EASD). Diabetologia.

[53] Tiano SM, Moczygemba LR, Hinds AJ, Jokerst J, Litten K, Roscoe CA (2025). Continuous glucose monitoring based glycemic control among underserved populations with type 2 diabetes at a health system of federally qualified health centers. Japha Pract Innov.

