# Supplementary material for: Transforming hypoglycemia prediction in adult type 1 diabetes: a systematic review and meta-analysis for precision care
Source: Open Life Sci. 2026 May 11;21(1):20251325. doi: 10.1515/biol-2025-1325 (PMC13157319; doi:10.1515/biol-2025-1325)
Supplement: Supplementary file 2 — Supplementary Material [file j_biol-2025-1325_suppl_002.pdf]

NIHR | National Institute for Health and Care Research

PROSPERO
International prospective register of systematic reviews

A systematic review of a model for predicting the risk of hypoglycemia in adults with type 1 diabetes mellitus

Qiang Zhang, Haojie Zhou

Citation
Qiang Zhang, Haojie Zhou. A systematic review of a model for predicting the risk of hypoglycemia in adults with type 1 diabetes mellitus. PROSPERO 2025 CRD420251012275. Available from https://www.crd.york.ac.uk/PROSPERO/view/CRD420251012275.

REVIEW TITLE AND BASIC DETAILS

Review title

A systematic review of a model for predicting the risk of hypoglycemia in adults with type 1 diabetes mellitus

Condition or domain being studied

Type 1 Diabetes Mellitus; Hypoglycemia; Predictable Behavior

P: Adults with type 1 diabetes mellitus.

I: Hypoglycemia risk prediction models .

C: No prediction models.

O: Prediction performance metrics

Rationale for the review

Hypoglycemia is a critical and potentially life-threatening complication of type 1 diabetes mellitus (T1DM), with adults experiencing an estimated 2–4 episodes of symptomatic hypoglycemia weekly and 1–2 episodes of severe hypoglycemia annually. Despite advancements in glucose monitoring and insulin therapy, hypoglycemia remains a significant barrier to achieving optimal glycemic control, contributing to morbidity, mortality, and reduced quality of life. Prediction models capable of identifying individuals at heightened risk of hypoglycemia could enable proactive interventions, personalized treatment adjustments, and targeted patient education, thereby minimizing preventable harm.

Existing hypoglycemia prediction models vary widely in methodology, incorporating clinical parameters, behavioral factors (e.g., insulin adherence, dietary patterns), and technological inputs . However, the evidence base supporting these models is fragmented, with inconsistencies in predictive performance metrics and limited head-to-head comparisons. Moreover, many models lack external validation or clinical translation, leaving clinicians uncertain about their utility in real-world settings. This systematic review aims to address these gaps by synthesizing the current state of hypoglycemia risk prediction models in adult T1DM populations. By evaluating the methodological rigor, predictive accuracy, and clinical applicability of existing models, this review will identify strengths, weaknesses, and opportunities for improvement. It will also highlight disparities in model performance across diverse subgroups (e.g., older adults, individuals with comorbidities) and technological platforms (e.g., CGM vs. intermittent capillary glucose testing). Ultimately, this synthesis will provide a foundation for evidence-based recommendations to guide clinical practice, inform the development of next-generation prediction tools, and prioritize research directions that enhance hypoglycemia prevention in T1DM.

Review objectives

To systematically evaluate the methodological rigor, predictive performance, and clinical utility of hypoglycemia risk prediction models in adults with type 1 diabetes, identify determinants of model accuracy, synthesize evidence to address research gaps, and provide recommendations for integrating validated tools into clinical practice.

Keywords

Type 1 diabetes; Adults; Prediction model; Hypoglycemia

Country

China

ELIGIBILITY CRITERIA

Population

Included

Adults ≥18 years of age with confirmed type 1 diabetes mellitus (T1DM).

Excluded

Pediatric populations (<18 years).

Intervention(s) or exposure(s)

Included

Hypoglycemia risk prediction models (clinical, technological, or algorithmic) designed to estimate risk of hypoglycemia .

Excluded

Non-predictive tools.

Comparator(s) or control(s)

This review does not have any comparators

Study design
Both randomized and nonrandomized study types will be included.

Included
Original research studies (observational, interventional, or modeling studies) evaluating hypoglycemia risk prediction models in adults with T1DM.

Validation studies, comparative effectiveness analyses, or technology-based assessments .Published in peer-reviewed journals.

Excluded
Case reports, editorials, letters, or reviews without original data.

Modeling studies lacking validation in adult T1DM populations.

Context
Exclusion Criteria

Study Type:

Case reports, editorials, letters, or reviews without original data.

Modeling studies lacking validation in adult T1DM populations.

Population:

Pediatric populations (<18 years).

Studies primarily including type 2 diabetes mellitus (T2DM) or mixed diabetes cohorts.

Intervention:

Non-predictive tools (e.g., general diabetes management apps without hypoglycemia risk estimation).

Outcome:

No reported prediction performance metrics or hypoglycemia-specific outcomes.

Other:

Duplicate publications or conference abstracts without full-text availability.

Studies conducted in non-human or animal models.

TIMELINE OF THE REVIEW

Date of first submission to PROSPERO
16 March 2025

Review timeline
Start date: 16 March 2025. End date: 16 March 2026.

Date of registration in PROSPERO
16 March 2025

AVAILABILITY OF FULL PROTOCOL

Availability of full protocol

A full protocol has been written and uploaded to PROSPERO. The protocol will be made available after the review is completed.

SEARCHING AND SCREENING

Search for unpublished studies
Only published studies will be sought.

Main bibliographic databases that will be searched
The main databases to be searched are *ASSIA - Applied Social Sciences Index & Abstracts*, *CENTRAL - Cochrane Central Register of Controlled Trials*, *CINAHL - Cumulative Index to Nursing and Allied Health Literature*, *CLIB - The Cochrane Library*, *Embase - Embase via Ovid*, *PubMed* and *SCI - Science Citation Index*.

Search language restrictions
The review will only include studies published in English and Chinese.

Search date restrictions
Databases will be searched for articles published before 30 May 2025, there are no restrictions on search start date.

Other methods of identifying studies
Other studies will be identified by: *contacting authors or experts* and *looking through all the articles that cite the papers included in the review ("snowballing")*.

Link to search strategy
A full search strategy has been uploaded to PROSPERO. The PDF may be accessed through this link https://www.crd.york.ac.uk/PROSPEROFILES/67be174275ff562c3f4211fcf5c8b97f.pdf.

Selection process
Studies will be screened independently by at least two people (or person/machine combination) with a process to resolve differences.

Other relevant information about searching and screening
None

DATA COLLECTION PROCESS

Data extraction from published articles and reports
Data will be extracted independently by at least two people (or person/machine combination) with a process to resolve differences.

Authors will be asked to provide any required data not available in published reports.

Study risk of bias or quality assessment
Risk of bias will be assessed using: *Cochrane RoB-2*, *PROBAST* and *ROBIS*

Data will be assessed independently by at least two people (or person/machine combination) with a process to resolve differences.

Additional information will be sought from study investigators if required information is unclear or unavailable in the study publications/reports.

Reporting bias assessment
Risk of bias due to missing results will be assessed

Certainty assessment
To evaluate the certainty (or confidence) in the evidence generated by this systematic review, we will employ the **Grading of Recommendations Assessment, Development, and Evaluation (GRADE)** framework. GRADE systematically assesses the quality of evidence across five domains and provides a transparent approach to grading confidence in estimates of effect or prediction model performance. Below is the structured application of GRADE to this review:

OUTCOMES TO BE ANALYSED

Main outcomes
The systematic review will prioritize the following outcomes to evaluate hypoglycemia risk prediction models in adults with type 1 diabetes mellitus (T1DM):

1. Prediction Model Performance Metrics

2. Hypoglycemia-Specific Outcomes

3. Clinical Impact Outcomes4. Model Characteristics

5. Adverse Events

6. Subgroup-Specific Outcomes

Additional outcomes
There are no additional outcomes.

PLANNED DATA SYNTHESIS

Strategy for data synthesis
To synthesize evidence on hypoglycemia risk prediction models in adults with type 1 diabetes, data will be combined using quantitative (meta-analysis) and qualitative (narrative synthesis) approaches, tailored to study homogeneity and outcome type.

CURRENT REVIEW STAGE

Stage of the review at this submission

Review stage	Started	Completed
Pilot work	✓	
Formal searching/study identification		
Screening search results against inclusion criteria		
Data extraction or receipt of IPD		

Review stage	Started	Completed
Risk of bias/quality assessment		
Data synthesis		

Review status
The review is currently planned or ongoing.

Publication of review results
Results of the review will be published in English and Chinese.

REVIEW AFFILIATION, FUNDING AND PEER REVIEW

Review team members
Mr Qiang Zhang (review guarantor and contact) Dali University. China.

No conflict of interest declared.

Mr Haojie Zhou. Dali University. China.

No conflict of interest declared.

Named contact
Mr Qiang Zhang (838769741@qq.com). Dali University. China.

Review affiliation
School of Nursing, Dali University, Yunnan Province, China

Funding source
Grant number
2025Y1186

Additional non-commercial funding information
Science Research Fund Project of Yunnan Provincial Department of Education, China (grant numbers 2025Y1186)

Additional commercial funding information
Science Research Fund Project of Yunnan Provincial Department of Education, China (grant numbers 2025Y1186)

Peer review
There has been no peer review of this planned review.

ADDITIONAL INFORMATION

Review conflict of interest
Declared individual interests are recorded under team member details. This review is funded by a commercial organisation.. No additional interests are recorded for this review.

Medical Subject Headings
Diabetes Mellitus, Type 1; Hypoglycemia

Check for similar records already in PROSPERO

PROSPERO identified a number of existing PROSPERO records that were similar to this one (last check made on 16 March 2025). These are shown below along with the reasons given by that the review team for the reviews being different and/or proceeding.

- Hypoglycemia risk prediction model for diabetes mellitus: a systematic review and critical appraisal [published 13 September 2021] [CRD42021272941]. The review was judged **not to be similar**
- Risk prediction models for hypoglycemia in diabetes patients: a systematic review [published 1 February 2021] [CRD42021232959]. The review was judged **not to be similar**
- Systematic evaluation of a predictive model for maternal neonatal hypoglycemia risk in gestational diabetes mellitus [published 2 February 2025] [CRD42025641818]. The review was judged **not to be similar**

PROSPERO version history

- Version 1.0, published 16 Mar 2025

Disclaimer

The content of this record displays the information provided by the review team. PROSPERO does not peer review registration records or endorse their content.

PROSPERO accepts and posts the information provided in good faith; responsibility for record content rests with the review team. The guarantor for this record has affirmed that the information provided is truthful and that they understand that deliberate provision of inaccurate information may be construed as scientific misconduct.

PROSPERO does not accept any liability for the content provided in this record or for its use. Readers use the information provided in this record at their own risk.

Any enquiries about the record should be referred to the named review contact
